# Determinants of Health-Related Quality of Life in School-Aged Children: A General Population Study in the Netherlands

**DOI:** 10.1371/journal.pone.0125083

**Published:** 2015-05-01

**Authors:** Marieke Houben-van Herten, Guannan Bai, Esther Hafkamp, Jeanne M. Landgraf, Hein Raat

**Affiliations:** 1 Socio-economic and Spatial Statistics, Statistics Netherlands, Heerlen, the Netherlands; 2 Department of Public Health, University Medical Centre Rotterdam, Rotterdam, the Netherlands; 3 HealthActCHQ, Boston, Massachusetts, United States of America; Instituto de Higiene e Medicina Tropical, PORTUGAL

## Abstract

**Background:**

Health related quality of life is the functional effect of a medical condition and/or its therapy upon a patient, and as such is particularly suitable for describing the general health of children. The objective of this study was to identify and confirm potential determinants of health-related quality of life in children aged 4-11 years in the general population in the Netherlands. Understanding such determinants may provide insights into more targeted public health policy.

**Methods:**

As part of a population based cross sectional study, the Child Health Questionnaire (CHQ) Parental Form 28 was used to measure health-related quality of life in school-aged children in a general population sample. Parents of 10,651 children aged 4-11 years were interviewed from January 2001 to December 2009.

**Results:**

Multivariate and regression analyses demonstrated a declined CHQ Physical Summary score for children who had >1 conditions, disorders or acute health complaints and who were greater consumers of healthcare; children with a non-western immigrant background; and children whose parents did not work. Lower CHQ Psychosocial Summary score was reported for children who had >1 conditions, disorders or acute health complaints, boys, children of single parents and obese children.

**Conclusion:**

The best predictors of health-related quality of life are variables that describe use of health care and the number of disorders and health complaints. Nonetheless, a number of demographic, socio-economic and family/environmental determinants contribute to a child’s health-related quality of life as well.

## Introduction

Good health is something all parents want for their children as it contributes to their happiness and well-being [1]. Health-related quality of life (HRQOL) is the functional effect of a medical condition and/or its therapy upon a patient [2,3]. It is thus subjective and multidimensional, encompassing physical and occupational function, psychological state, social interaction and somatic sensation [3]. It is therefore, particularly suitable for describing the health of children in a general representative sample that may also include specific condition groups [4–7].

Previous studies have shown that many factors are associated with HRQOL in children. Logically, variables correlating with bad health, e.g., the number of conditions or health problems [8] or more indirectly the number of health care visits [9], are negatively associated with HRQOL. Additional studies have examined the potential demographic and/or socioeconomic determinants of diminished HRQOL. Girls tend to have lower HRQOL than boys [9–12]. HRQOL declines with age [10–12], although sometimes this was found to be more distinctive for girls [11]. Also, a low socioeconomic position of the child or the child’s family, as measured by income [12], parental education level or family wealth [13], negatively influences parental reports of child HRQOL. In addition, children living in neighbourhoods scoring high on satisfaction to live there and on good access to services like recreational programmes and stores with fresh fruit and vegetables reported higher HRQOL [14].

Most studies mentioned examined different and limited numbers of potential determinants. Thus, findings from each of these separate studies need to be combined to generate an overall understanding of potential determinants of HRQOL. Such efforts are hampered because of differences in key study components such as overall design, data collection methodology child age and use of differing HRQOL survey instruments. The current study avails the opportunity to further assess identified determinants in a more robust and potential diverse population—i.e., not a community or clinical sample, but a very large randomly selected sample drawn from national data, and therefore generalizable. It includes not only known variables such as gender, age, socioeconomic position, and health but also variables that, to our knowledge, have not been addressed before such as cultural/ethnic differences and family composition. To assess HRQOL, the Child Health Questionnaire short-form (CHQ-PF28) was used.

The CHQ-PF28 focusses on the health-related part of quality of life, so we expect that the strongest determinants are factors that directly or indirectly describe the child’s health. That is, the number of chronic conditions or health complaints, or the use of health care, which may be considered as manifestation of acute health problems or limitations. We expect only minor effects for determinants like demographic, socio-economic or family/environmental variables.

## Methods

### Data source

The study data source was the national Dutch Health Interview Survey (DHIS), conducted by Statistics Netherlands, using trained in-house interviewers. The DHIS is a cross-sectional survey, conducted yearly, amongst the Dutch population living in non-institutionalised households. Each month, a stratified two-step-sample of persons is taken from the Dutch Municipal Personal Records. The yearly response rate of the age group 4–11 years is approximately 75%. For this study, a 9 year set of surveys was used. For respondents aged 0–11 years, one parent participates in the interview. Between January 2001 and December 2009, the parents of 10,651 children aged 4–11 years were interviewed. The mean age was 7.47 years (SD = 2.29), 49.1% were girls. Data was weighted to take into account the person’s probability of selection and to compensate for (selective) nonresponse. By so doing, responses are adjusted to the actual distribution of persons in the target population, allowing generalization at the national level. The weighting model included sex, age, marital status, regional information (province, part of the country, urbanization), household size, ethnicity and interview month.

Parents received written study information and participation was elective. According to Dutch law (Wet medisch wetenschappelijk onderzoek met mensen), formal consent (e.g., from a medical ethics committee) was not required as this study relied on secondary anonymised data collection in the context of performing statutory tasks. Data collection and processing was in strict accordance with the national standard. At no time did the datasets contain direct identifiers.

### Questionnaire

The Dutch version of the parent-completed CHQ-PF28 was administered via structured interview as part of the larger DHIS interview. This CHQ measure was selected because it has been rigorously translated into 78 languages (http://www.healthactchq.com/chq-t.php) and specifically evaluated for use in the Netherlands in very young children [15,16] and is easy to administer in large population studies [4]. The CHQ-PF28 includes multi-item Likert-type scales and global items that assess 14 unique physical and psychosocial concepts. Per published instructions [4], a mean scale score is derived and items are then standardized on a 0–100 continuum with a higher score representing better HRQOL. Scores can also be combined to derive a two component summary—the CHQ Physical (PhS) and Psychosocial Summary (PsS) Scales. The CHQ Summaries are based on factor weights from a US representative sample of children ages 5–18 years of age [4]. A score of 50 represents the mean of the US reference population sample and the standard deviation is ten points above/below the mean [4]. The weighted US values to derive 2 component summary scales (PhS and PsS) have been used with success in both the aforementioned Dutch and other international studies [17,18]. See Table 1 for a description /interpretation of scales.

**Table 1:** CHQ-PF28 scales, number of items per scale, and score interpretation. ^a^

Scale	Nr of Items	Description low score	Description high score
**Physical functioning (PF)**	3	Child is limited a lot in performing all physical activities, including self-care, because of health	Child performs all types of physical activities, including the most vigorous, without limitations attributable to health
**Role functioning: emotional/behaviour (REB)**	1	Child is limited a lot in school work or activities with friends as a result of emotional or behavioural problems	Child has no limitations in schoolwork or activities with friends as a result of emotional or behavioural problems
**Role functioning: physical (RP)**	1	Child is limited a lot in school work or activities with friends as a result of physical health	Child has no limitations in school work or activities with friends as a result of physical health
**Bodily pain (BP)**	1	Child has extremely severe, frequent, and limiting bodily pain	Child has no pain or limitations because of pain
**General behaviour (BE)**	4	Child very often exhibits aggressive, immature, delinquent behaviour	Child never exhibits aggressive, immature, delinquent behaviour
**Mental health (MH)**	3	Child has feelings of anxiety and depression all of the time	Child feels peaceful, happy, and calm all of the time
**Self-esteem (SE)**	3	Child is very dissatisfied with abilities, looks, family/peer relationships, and life overall	Child is very satisfied with abilities, looks, family/peer relationships, and life overall
**General health perceptions (GH)**	4	Parent believes child's health is poor and likely to get worse	Parent believes child's health is excellent and will continue to be so
**Parental impact: emotional (PE)**	2	Parent experiences a great deal of emotional worry/concern as a result of child's physical and/or psychosocial health	Parent doesn't experience feelings of emotional worry/concern as a result of child's physical and/or psychosocial health
**Parental impact: time (PT)**	2	Parent experiences a lot of limitations in time available for personal needs because of child's physical and/or psychosocial health	Parent doesn't experience limitations in time available for personal needs because of child's physical and/or psychosocial health
**Family activities (FA)**	2	The child's health very often limits or interrupts family activities or is a source of family tension	The child's health never limits and interrupts family activities or is a source of family tension
**Family cohesion (FC)**	1	Family's ability to get along is rated 'poor'	Family's ability to get along is rated 'excellent'
**Change in health (CH)**	1	Child's health is much worse now than one year ago	Child's health is much better now than one year ago

a. From the CHQ manual [2]. Reproduced with permission from JM Landgraf.

### Description of determinants

The potential determinants are listed in Table 2 and were selected a priori based on literature study and their availability in the current DHIS dataset. Ethnicity (western immigrant, non-western immigrant and native Dutch people) and household level of income were identified from separate databases (Dutch Municipal Personal Records and Dutch Tax Authorities, respectively). Children whose parents were born outside the Netherlands were identified as immigrants (even if the child was of Dutch nationality). Western immigrants originated from Europe (excluding Turkey), North America, Oceania, Indonesia or Japan. Non-western immigrants originated from Africa, South America, Asia (excluding Indonesia and Japan) or Turkey. Low income households had an income below the Dutch low income threshold using the Dutch supplementary benefit level. “Urbanization” of the child’s primary place of residence was defined as the average number of addresses per square kilometre within a one kilometre radius. For exact boundaries see http://www.cbs.nl/en-GB/menu/methoden/toelichtingen/alfabet/u/urbanisation-rate.htm.

**Table 2:** General characteristics of the study population.

Variable	Levels	PhS		PsS	
		N	%	N	%
**Gender**	**Male**	4227	51.2%	4258	50.9%
	**Female**	4033	48.8%	4108	49.1%
**Age**	**4**	1024	12.4%	1054	12.6%
	**5**	1048	12.7%	1078	12.9%
	**6**	1038	12.6%	1055	12.6%
	**7**	1038	12.6%	1046	12.5%
	**8**	1041	12.6%	1039	12.4%
	**9**	1043	12.6%	1046	12.5%
	**10**	1000	12.1%	1011	12.1%
	**11**	1028	12.4%	1037	12.4%
**Ethnicity**	**Native Dutch people**	5924	79.8%	5985	79.7%
	**Immigrants, Western**	431	5.8%	440	5.9%
	**Immigrants, Non-west.**	1066	14.4%	1080	14.4%
**Urbanisation rate**	**Very high**	1395	16.9%	1415	16.9%
	**High**	2213	26.8%	2232	26.7%
	**Moderately high**	1675	20.3%	1698	20.3%
	**Low**	1853	22.4%	1873	22.4%
	**Very low**	1124	13.6%	1147	13.7%
**Single parent family**	**Two parent family**	7402	89.7%	7501	89.8%
	**Single parent family**	849	10.3%	856	10.2%
**Siblings in household**	**Only child**	865	10.5%	870	10.4%
	**1 brother or sister**	4245	51.5%	4297	51.4%
	**More brothers/sisters**	3140	38.1%	3187	38.1%
**Working situation parents** ^**a**^	**Both parents work**	5173	69.3%	5230	69.2%
	**One parent works**	1829	24.5%	1858	24.6%
	**Parents do not work**	466	6.2%	473	6.3%
**Highest parental educational level** ^**b**^	**Low**	437	7.5%	434	7.4%
	**Medium**	2883	49.6%	2909	49.5%
	**High**	2495	42.9%	2532	43.1%
**Low household income**	**No**	7068	88.7%	7154	88.7%
	**Yes**	900	11.3%	913	11.3%
**Parents' smoking behaviour** ^**c**^	**Both parents smoke**	939	13.1%	945	13.0%
	**One parent smokes**	2072	28.8%	2090	28.8%
	**Parents don't smoke**	4173	58.1%	4234	58.2%
**BMI child**	**Normal weight**	4885	83.1%	4934	83.0%
	**Overweight**	751	12.8%	760	12.8%
	**Obese**	242	4.1%	250	4.2%
**Nr of chronic conditions**	**None**	6475	78.4%	6496	77.6%
	**1**	1461	17.7%	1506	18.0%
	**2 or more**	324	3.9%	364	4.4%
**Nr of behavioural /learning disorders**	**None**	7528	91.2%	7663	91.6%
	**1**	651	7.9%	632	7.6%
	**2 or more**	78	0.9%	68	0.8%
**Nr of acute health Complaints**	**None**	5001	60.6%	4997	59.8%
	**1**	2010	24.4%	2037	24.4%
	**2**	941	11.4%	983	11.8%
	**3 or more**	294	3.6%	335	4.0%
**Visited GP last 14 days**	**No**	7675	92.9%	7725	92.3%
	**Yes**	585	7.1%	641	7.7%
**Visited a medical specialist last 14 days**	**No**	7984	96.6%	8063	96.4%
	**Yes**	277	3.4%	303	3.6%
**Used prescription medicines last 14 days**	**No**	7092	85.9%	7133	85.3%
	**Yes**	1168	14.1%	1232	14.7%
**Used non-prescription medicines last 14 days**	**No**	5960	72.2%	5996	71.7%
	**Yes**	2299	27.8%	2369	28.3%
**Hospitalisation last year**	**No**	8121	98.3%	8211	98.1%
	**Yes**	140	1.7%	155	1.9%

a. If a parent in a single family works, he/she is included in the category ‘Both parents work’.

b. Parental education is missing for some years.

c. If a parent in a single family smokes, he/she is included in the category ‘One parent smokes’.

Parents were asked to indicate if their child ever had cancer, or experienced health or behavioural issues during the previous 12 months: congenital defects, diabetes, migraine/ severe headache, asthma, psoriasis, eczema, arthritis/rheumatism, severe/protracted disorders of the intestines, back, neck/shoulder, arm or hand; dyslexia, intellectual disability, and presence of at least three core ADHD symptoms (DSM-criteria: restless behaviour/not being able to sit still, fidgeting/squirming, short attention span). An open-ended question about any other chronic conditions and behavioural issues not mentioned was also included. The occurrence of headache, tiredness, back, muscle and joint pains during the last 14 days were used to determine “number of health complaints”. Body-Mass Index (BMI) was calculated using child’s height/weight as reported by the parent. International age- and sex specific boundaries [19] were used to define weight categories (normal weight, overweight, obese).

### Statistical Analyses

Analyses were performed using SPSS 14.0. Outliers (values above/below 3xSD +/- mean) were deleted. Non-response was compensated using weights [20]. Bivariate analyses were performed to assess differences in the two CHQ Summaries between (independent) groups of children. Distributions of the summary scores were somewhat negatively skewed (PhS: Skewness = -1.85, se =. 027. PsS: Skewness = -.65, se =. 027). Because of the large number of respondents, however, parametric tests are preferred to nonparametric alternatives [21]. Oneway ANOVA’s were used; a p-value <0.05 was considered to be statistically significant. In case of a significant effect, post-hoc Tukey HSD analyses were performed. Clinical significance was assessed using effect size which was estimated by dividing the difference in mean scores between subgroups by the largest SD and interpreted by using Cohen’s effect sizes (d):. 2 ≤ d <. 5 small difference,. 5 ≤ d <. 8 moderate, and d ≥. 8 large [22]. In order to interpret the effect size in real-world terms, the minimum important difference (MID) was used [23–25]. In most circumstances, the threshold of discrimination for changes in HRQOL for chronic diseases appears to be approximately half a standard deviation (SD) [26,27].

Stepwise multivariate linear regression was performed to identify determinants that could best explain the two CHQ-PF28 Summary Scale means. Variables were entered independently, commencing with the highest F-value as determined by the bivariate analyses. The procedure was repeated until the addition of another independent variable did not increase the explained variation (adjusted R-square) or until the variable included was not statistically significant. Thus, the final models included only those variables that were statistically significant and enhanced the degree of explanation. Multicollinearity was checked.

## Results

General characteristics of the study population (N = 10,651, mean age of 7.47 years, 49.1% were girls), can be found in Table 2. Mean scores for several age and gender groups are presented exclusively for illustration purposes in S1 Table.

### Bivariate Analyses

Table 3 provides CHQ mean scores, standard deviations, F-values, p-values and effect sizes of the tested determinants of the CHQ Summary Scores. Summary Scores were not calculated for 3.1% of the cases (327 of 10,651) because one or more items were missing or answered ‘don’t know’.

**Table 3:** Bivariate associations with CHQ-score and effect sizes based on interview administration methods. ^#^

Variable	Levels	PhS		PsS	
		mean (F-value, p-value)	sd *effect size*	mean (F-value, p-value)	sd *effect size*
**Gender**	**Male**	57.04	6.22	52.68	6.66
	**Female**	56.95	6.04	53.36	6.29
		(<1,. 51)	*0*.*01*	(22.93, <.001)*	*0*.*10*
**Age**	**4**	56.63	6.47	53.30	6.12
	**5**	56.86	6.33	53.75	6.17
	**6**	57.42	5.90	53.06	6.28
	**7**	57.03	6.09	53.21	6.54
	**8**	57.09	5.92	52.49	6.64
	**9**	57.20	5.96	52.65	6.73
	**10**	56.73	6.33	52.70	6.78
	**11**	57.02	6.01	52.88	6.58
		(<2,. 09)	*0*.*12*	(4.26, <.001)*	*0*.*19*
**Ethnicity**	**Native Dutch people**	57.15	6.11	53.10	6.42
	**Immigrants, Western**	56.50	6.52	52.68	6.72
	**Immigrants, Non-western**	56.35	6.31	52.68	6.60
		(9.13, <.001)*	*0*.*12*	(<2.5,. 08)	*0*.*06*
**Urbanisation rate**	**Very high**	56.58	6.17	52.89	6.45
	**High**	56.96	6.09	53.04	6.55
	**Moderately high**	56.98	6.29	52.89	6.46
	**Low**	57.18	6.09	52.99	6.48
	**Very low**	57.32	5.98	53.32	6.49
		(2.82,. 02)*	*0*.*12*	(<1,. 44)	*0*.*07*
**Single parent family**	**Two parent family**	57.05	6.09	53.24	6.38
	**Single parent family**	56.60	6.47	50.95	7.07
		(4.01,. 04)*	*0*.*07*	(96.81, <.001)*	*0*.*32* ^*a*^
**Siblings in household**	**Only child**	52.00	7.76	52.53	6.80
	**Has 1 brother or sister**	52.60	7.12	53.00	6.42
	**Has more brothers/sisters**	52.76	7.20	53.15	6.49
		(3.81,. 02)*	*0*.*1*	(3.11,. 04)*	*0*.*09*
**Working situation Parents**	**Both parents work**	57.17	6.01	53.13	6.35
	**One parent works**	56.90	6.32	53.09	6.48
	**Parents do not work**	55.53	6.98	51.63	7.25
		(15.62, <.001)*	*0*.*24* ^*a*^	(11.78, <.001)*	*0*.*21* ^*a*^
**Highest parental educational level**	**Low**	56.63	6.20	52.20	6.87
	**Medium**	57.19	6.16	52.96	6.52
	**High**	57.02	5.95	53.52	6.22
		(<2,. 17)	*0*.*09*	(10.30, <.001)*	*0*.*19*
**Low household income**	**No**	57.04	6.10	53.17	6.39
	**Yes**	56.80	6.20	52.37	6.82
		(<1.5,. 27)	*0*.*04*	(12.71, <.001)*	*0*.*12*
**Parents' smoking behaviour**	**Both parents smoke**	57.05	6.16	52.82	6.49
	**One parent smokes**	56.75	6.36	52.71	6.59
	**Parents do not smoke**	57.12	6.08	53.22	6.38
		(<2.5,. 08)	*0*.*06*	(4.84,. 01)*	0.08
**BMI child**	**Normal weight**	57.06	6.08	53.12	6.46
	**Overweight**	56.68	6.33	52.90	6.57
	**Obese**	55.86	6.31	51.49	7.02
		(5.37,. 005)*	*0*.*19*	(7.65, <.001)*	*0*.*23* ^*a*^
**Nr of chronic conditions**	**None**	57.77	5.47	53.27	6.25
	**1**	55.04	6.96	52.41	6.98
	**2 or more**	50.36	8.27	50.80	7.92
		(341.50, <.001)*	*0*.*90* ^*c*^	(33.19, <.001)*	*0*.*31* ^*a*^
**Nr of behavioural /learning disorders**	**None**	57.05	6.02	53.45	6.21
	**1**	56.68	6.93	48.58	7.41
	**2 or more**	54.33	8.94	44.42	7.17
		(8.63, <.001)*	*0*.*30* ^*a*^	(237.35, <.001)*	*1*.*26* ^*c*^
**Number of acute health complaints**	**None**	58.44	4.79	53.86	6.07
	**1**	55.93	6.45	52.48	6.63
	**2**	53.70	7.59	51.53	7.25
	**3 or more**	50.51	8.67	50.94	7.58
		(353.60, <.001)*	*0*.*92* ^*c*^	(52.07, <.001)*	*0*.*36* ^*a*^
**Visited a GP last 14 days**	**No**	57.35	5.77	53.02	6.43
	**Yes**	52.41	8.49	52.84	7.20
		(367.28, <.001)*	*0*.*58* ^*b*^	(<1,. 49)	*0*.*03*
**Visited medical specialist last 14 days**	**No**	57.15	5.97	53.05	6.44
	**Yes**	52.65	8.57	52.06	7.69
		(146.70, <.001)*	*0*.*53* ^*b*^	(6.69,. 01)*	*0*.*13*
**Used prescription medicines last 14 days**	**No**	57.60	5.60	53.16	6.31
	**Yes**	53.32	7.74	52.14	7.42
		(519.34, <.001)*	*0*.*55* ^*b*^	(26.03, <.001)*	*0*.*14*
**Used non pre-scription medicines last 14 days**	**No**	57.86	5.39	53.24	6.34
	**Yes**	54.77	7.27	52.41	6.83
		(441.49, <.001)*	*0*.*42* ^*a*^	(27.85, <.001)*	*0*.*12*
**Hospitalization last year**	**No**	57.07	6.07	53.02	6.47
	**Yes**	52.88	8.03	52.45	7.62
		(64.39, <.001)*	*0*.*52* ^*b*^	(<1.5,. 28)	*0*.*07*

Effect sizes are highest vs. lowest mean CHQ-score.

a = small difference,

b = moderate difference,

c = large difference.

# Data were calculates using US based weights and are provided for illustrative purposes. Not for general use.

* Statistically significant.

The number of chronic conditions or number of acute health complaints (i.e, health problems), were associated with lower observed scores for both the CHQ PhS *and* PsS Summary Scales (p<.001). Large effect sizes (d) were found for: the PhS score in children who had ≥3 health complaints (e.g., headache /tiredness)(d = 0.90, p<.001); the PsS score in children with ≥2 chronic conditions (d = 0.92, p<.001); and the PsS score in children with ≥2 reported behavioural or learning disorders (d = 1.26, p<.001), all compared to children without such disorders (Table 3). The variables with moderate or large effect sizes (d≥.5) also met the criterion of the minimal important difference, i.e. a difference of half a SD.

### Regression Analyses

For the PhS, the final multivariate regression model included: ethnicity, parent work status, number of chronic conditions, behavioural/learning disorders, health complaints, general practitioner and/or medical specialist consultations, medication status and hospitalization. The adjusted R-square for the final model was. 24. For the PsS, the final regression model included: gender, single parent family, obesity, number of chronic conditions, behavioural/learning disorders, and health complaints. The adjusted R-square for the final model was. 08. See Table 4 for the coefficients and corresponding confidence intervals.

**Table 4:** Multivariate analysis of CHQ-PF28 scores.

Variable	Physical Summary scale			Psychosocial Summary scale		
	coefficient (N = 7171)	95% confidence interval		coefficient (N = 8340)	95% confidence interval	
		*lower bound*	*upper bound*		*lower bound*	*upper bound*
**Gender (male = Ref)**				0.51*	0.22	0.80
**Ethnicity (native Dutch = Ref)**	-0.63^+^	-1.00	-0.25			
**Single parent family (Two parent = Ref)**				-0.96*	-1.20	-0.71
**Number of working parents**	0.32^+^	0.10	0.53			
**Obesity (no obesity = Ref)**				-1.53*	-2.31	-0.74
**Number of chronic conditions**	-2.00*	-2.25	-1.76	-0.50*	-0.75	-0.24
**Number of behavioural/learning disorders**	0.44^+^	0.06	0.82	-4.29*	-4.74	-3.84
**Number of acute health complaints**	-1.85*	-2.00	-1.69	-0.92*	-1.09	-0.75
**Visited a general practitioner last 14 days**	-2.89*	-3.39	-2.39			
**Visited a medical specialist last 14 days**	-3.33*	-4.01	-2.65			
**Used prescription medicines last 14 days**	-2.01*	-2.40	-1.61			
**Used non-prescription medicines last 14 days**	-1.71*	-2.01	-1.42			
**Hospitalization last year**	-2.86*	-3.82	-1.90			

Ref indicates a category used as a standard reference. If there is no reference, the variable is treated as a continuous variable in the model.

* p <. 001

+ p <. 005.

## Discussion

For policy makers, understanding the variables that determine children’s HRQOL can provide insight into developing more targeted public health policies. In this study, we examined a large range of determinants of HRQOL, i.e., demographic, socio-economic, health and family/environmental, in a national school-aged Dutch sample using the CHQ-PF28. The bivariate analyses have mapped the different determinants. The multivariate regression analyses allowed for an independent comparison of the determinants to identify the most defining ones.

As expected, and as was reported previously by others [8], conducting bivariate analyses, large clinically significant differences for both CHQ-PF28 PhS and PsS Summaries were observed for the number of parent-reported health conditions/disorders/complaints. Moderate differences were found for “use of health care” (consulting a GP or medical specialist, use of prescribed medication, hospitalization). Hence, the best predictors in the multivariate regression analysis are variables that describe the use of health care and the number of chronic conditions and health complaints for PhS and the number of behavioural/learning disorders for PsS. This difference is understandable given that the “psychosocial CHQ scales” (e.g., mental health, behaviour, self-esteem) load more substantially on the PsS. For PsS, health care determinants do not contribute to the scale variance. The moderate and large effect sizes we found can be regarded as clinically important differences, i.e. they met the criterion of the minimum important difference (half a SD).

A number of demographic, social-economic and family/environmental determinants were found with small or even no clinical significance using bivariate analysis. However, some contributed significantly, although only slightly, in the regression analyses. This finding suggests that gender, ethnicity, parent work status, single parent family and obesity affected HRQOL independent of the number of chronic conditions or health issues.

For PhS, one important significant contributor was the non-western immigrant status. Several surveys conducted at the national and local level showed inequalities in health in non-western immigrant children compared to non-migrant children in the Netherlands [28].Children of non-working parents had a lower mean score, which had been reported in a previous study using the PedsQL [9]. The positive effect of working parents may be explained by the better family socio-economic position which theoretically can provide a more stimulating and healthier environment. A recent Dutch study showed that children from low socio-economic families experience more asthma symptoms, poorer general health, more frequent respiratory infections, and are more often overweight or obese [29]. Conversely, the child’s poor health—as perceived by the parent—may be the reason for the parent to stay at home, or reduce working hours. An effect in an unexpected direction was found for the number of behavioural/learning disorders. While bivariate analyses showed that parents reported lower PhS for children with at least one learning/behaviour disorder, multivariate regression analysis showed the opposite, to a small extent. We examined whether this is a consequence of the fact that our multivariate analyses included suppressive factors for low PhS. In the models, no multicollinearity was found and we could not identify a suppressor effect (data not shown). The relatively high PhS for children with a learning or behavioural disorder may be considered a chance finding.

For PsS, the best predictor among the non-health-related determinants was obesity. Several studies have shown that obese children have lower HRQOL than normal weight children [30–33]. In addition, parents of girls reported slightly higher scores. However, lower HRQOL has been reported for girls using the KIDSCREEN [34–37] and PedsQL [9]. This difference may be explained by the item content for the General Behaviour subscale which is weighted highly in the calculation of the PsS and which asks about frequency of aggressive/immature/delinquent behaviour (arguing, inability to concentrate, lying/cheating). Boys tend to employ direct means of aggression, whereas girls more often employ indirect, often less visible, means of aggression [35]. Also, most respondents (81%) were mothers and parental gender has been shown to be a mediating factor in the reporting of a child’s health [37]. Finally, living in single parent families was a significant contributor to PsS score variance: a lower mean score was observed for children living in a single parent family. This has been reported by others as well [37].

Collectively, these data suggest that a child’s HRQOL—as reported by the parent—is mainly dependent on the child’s health, and to a smaller extent on demographic, socioeconomic and family/environmental factors.

### Strengths and Limitations

Overall, this effort to explore the determinants of HRQOL in children extended the current literature by measuring a wider array of variables than previous studies and did so in a large, representative sample. Even still, the independent variables were limited to those found in the dataset DHIS (demographic, social-economic factors and parents’ reports of children’s medical care use and medical conditions), and explained only a small part of the variance of the CHQ-PF28 PhS and especially PsS. Although the result does not change how we might think about the factors that contribute to children’s HRQOL, they do confirm the role of various factors in a large, representative sample. Other important determinants were not captured and thus further study is needed. For example, early life experiences and maternal factors (gestation, health symptoms in pregnancy, anxiety and depression) were found to impact HRQOL [38] and Mansour et al. found that children’s perceived closeness to school personnel and the school environment are positively associated with HRQOL [9].

Other methodological considerations are warranted. First, although the CHQ-PF28 was developed for parents of children aged 5 years and older, the focus was on school-aged children, which in the Netherlands includes 4-year-olds. Previous work has demonstrated that the Dutch CHQ-PF28 can be successfully applied and validated among children aged 4–13 [15]. Further, data used in these analyses were gathered at home using face to face interviews. US factor weights to calculate the CHQ summaries were derived using paper-and-pencil methods. However, publications using the same data source (but from earlier years) and a school based sample demonstrated that the CHQ-PF28 is a feasible instrument in the Netherlands irrespective of administration [15,39]. Thirdly, both Cohen’s d and difference of half a SD were used for the interpretation of relevant differences in HRQOL. Although this is an accepted method [40] and helpful to interpret findings in real-world terms, there are still insufficient data to understand the relative impact of the observed score differences. Empirically defined cut-off points for minimal important differences for HRQOL measures such as the CHQ-PF28 are important in future research [41]. Finally, a cross-sectional design was applied with data that were collected during nine consecutive years; it is therefore possible that a time trend in the data could potentially confound findings. To determine if such was the case, additional bivariate analyses were performed to evaluate the impact of the variable ‘Year of data collection’ on the CHQ Physical and Psychosocial Summary Scale Scores (statistical significance and effect sizes were evaluated). Bivariate analyses showed that survey year did not significantly effect PhS (d = 0.07, p =. 888) or PsS (d = 0.10, p =. 390). Thus, the trend was not considered a serious threat to the overall findings.

## Supporting Information

S1 Table Illustration of age-gender groupings using interviewer administration. (DOCX)

## References

[1] Gerdtham UG, Johannesson M. The relationship between happiness, health, and socio-economic factors: results based on Swedish microdata. J Socio Econ. 2001;30: 553–7.

[2] Cella D. Measuring quality of life in palliative care. Seminars in Oncology. 1995;22:73–81. 7537908

[3] Schipper H, Clinch JJ, Olweny CLM. Quality of life studies: definitions and conceptual issues In: Spilker B, editor. Quality of Life and Pharmacoeconomics in Clinical Trials. Philadelphia: Lippincott-Raven Publishers;1996

[4] Landgraf JM, Abetz L, Ware JE. The Child Health Questionnaire (CHQ): A user's manual (2^nd^ printing) Boston: HealthAct; 1999.

[5] Landgraf JM. Practical considerations in the measurement of health-related quality of life in child/adolescent clinical trial In: Fayers P, Hays RD, 2nd edition editors. Assessing the Quality of life in Clinical Trials. Oxford: Oxford Press; 2004

[6] Landgraf JM. Health-related quality of life assessment in pediatric clinical trials: a brief introduction In: Helms P, Stonier P, editors. Paediatric Clinical Research Manual. Euromed Communications; 2005.

[7] Bullinger M. Assessing health related quality of life in medicine: an overview over concepts, methods and applications in international research. Restor Neurol Neurosci. 2002;20:93–101. 12454358

[8] Waters E, Davis E, Nicolas C, Wake M, Lo SK. The impact of childhood conditions and concurrent morbidities on child health and well-being. Child Care Health Dev. 2008;34:418–29. 10.1111/j.1365-2214.2008.00825.x 19154551

[9] Mansour ME, Kotagal U, Rose B, Ho M, Brewer D, Roy-Chaudhury A, et al Health-Related Quality of Life in Urban Elementary Schoolchildren. Pediatrics. 2003;111:1372–81. 12777555 10.1542/peds.111.6.1372

[10] Michel G, Bisegger C, Fuhr DC, Abel T, The KIDSCREEN group. Age and gender differences in health-related quality of life of children and adolescents in Europe: a multilevel analysis. Qual Life Res. 2009;18:1147–57. 10.1007/s11136-009-9538-3 19774493

[11] Ravens-Sieberer U, Gosch A, Rajmil L, Erhart M, Bruil J, Duer W, et al The KIDSCREEN-52 quality of Life Measure for Children and Adolescents: Psychometric results from a cross-cultural survey in 13 European countries. Value Health. 2007;11:645–58. 10.1111/j.1524-4733.2007.00291.x 18179669

[12] Simon AE, Chan KS, Forrest CB. Assessment of children's health-related quality of life in the United States with a multidimensional index. Pediatrics. 2008;121:118–26. 10.1542/peds.2007-0480 18056290

[13] Von Rueden A, Gosch LR, Bisegger C, Ravens-Sieberer U. Socioeconomic determinants of health related quality of life in childhood and adolescence: results from a European study. J Epidemiol Community Health. 2006;60:130–5. 16415261 10.1136/jech.2005.039792 PMC2566139

[14] Wu XY, Ohinmaa A, Veugelers PJ. Sociodemographic and neighbourhood determinants of health-related quality of life among grade-five students in Canada. Qual Life Res. 2010;19:969–76. 10.1007/s11136-010-9663-z 20446044

[15] Raat H, Botterweck AM, Landgraf JM, Hoogeveen WC, Essink-Bot ML. Reliability and validity of the short form of the child health questionnaire for parents (CHQ-PF28) in large random school based and general population samples. Epidemiol Community Health.2005;59:75–82. 15598731 10.1136/jech.2003.012914 PMC1763365

[16] Raat H, Landgraf JM, Bonsel GJ, Gemke RJ, Essink-Bot ML. Reliability and validity of the child health questionnaire-child form (CHQ-CF87) in a Dutch adolescent population Qual Life Res. 2002;11:575–81. 12206578 10.1023/a:1016393311799

[17] Beckung E, White-Koning M, Marcelli M, McManus V, Michelsen S, Parkes J, et al Health status of children with cerebral palsy living in Europe: a multi-centre study. Child Care Health. 2008;34:806–14. 10.1111/j.1365-2214.2008.00877.x 18959578

[18] Spurrier NJ, Sawyer MG, Clark JJ, Baghurst P. Socio-economic differentials in the health-related quality of life of Australian children: results of a national study. Aust N Z J Public Health. 2003;27:27–33. 14705264 10.1111/j.1467-842x.2003.tb00376.x

[19] Cole TJ, Flegal KM, Nicholls D, Jackson AA. Body mass index cut offs to define thinness in children and adolescents: international survey. BMJ. 2007;335:194 17591624 10.1136/bmj.39238.399444.55 PMC1934447

[20] Banning R, Camstra A, Knottnerus P. Sampling theory: Sampling design and estimation methods The Hague/Heerlen: Statistics Netherlands; 2012.

[21] Fagerland MW. T-tests, non-parametric tests, and large studies—a paradox of statistical practice? BMC Med Res Methodol. 2012;12:78 22697476 10.1186/1471-2288-12-78 PMC3445820

[22] Cohen J. Statistical power analysis for the behavioral sciences New York: Academic Press; 1977.

[23] Nichol MB, Epstein JD. Separating gains and losses in health when calculating the minimum important difference for mapped utility measures. Qual Life Res. 2008;17(6):955–61. 10.1007/s11136-008-9369-7 18615271

[24] Cole JC, Lin P, Rupnow MF. Minimal important differences in the Migraine-Specific Quality of Life Questionnaire (MSQ) version. Cephalalgia. 2009;29(11):1180–7. 19830883 10.1111/j.1468-2982.2009.01852.x

[25] Jaeschke R, Singer J, Guyatt GH. Measurement of health status.Ascertaining the minimal clinically important difference. Control Clin Trials. 1989;10(4):407–15. 2691207 10.1016/0197-2456(89)90005-6

[26] Masood M, Masood Y, Saub R, Newton JT. Need of minimal important difference for oral health-related quality of life measures. J Public Health Dent. 2014;74(1):13–20. 10.1111/j.1752-7325.2012.00374.x 22994869

[27] Norman GR 1, Sloan JA, Wyrwich KW. Interpretation of changes in health-related quality of life: the remarkable universality of half a standard deviation. Med Care. 2003;41(5):582–92. 12719681 10.1097/01.MLR.0000062554.74615.4C

[28] Schulpen TW. Migration and child health: the Dutch experience. Eur J Pediatr. 1996;155:351–6. 8741029 10.1007/BF01955260

[29] Ruijsbroek A, Wijga AH, Kerkhof M, Koppelman GH, Smit HA, Droomers M. The development of socio-economic health differences in childhood: results of the Dutch longitudinal PIAMA birth cohort. BMC Public Health. 2011;11:225 10.1186/1471-2458-11-225 21486447 PMC3094243

[30] Grieken van A, Veldhuis L, Renders CM, Landgraf JM, Hirasing RA, Raat H. Impaired parent-reported health-related quality of life of underweight and obese children at elementary school entry. Qual Life Res. 2013; 22:917–28. 10.1007/s11136-012-0211-x 22695828 PMC3636439

[31] Wake M, Salmon L, Waters E, Wright M, Hesketh K. Parent-reported health status of overweight and obese Australian primary school children: a cross-sectional population survey. Int J Obes Relat Metab Disord. 2002;26:717–24. 12032758 10.1038/sj.ijo.0801974

[32] Ottova V, Erhart M, Rajmil L, Dettenborn-Betz L, Ravens-Sieberer U. Overweight and its impact on the health-related quality of life in children and adolescents: results from the European KIDSCREEN survey. Qual Life Res. 2012;21:59–69. 10.1007/s11136-011-9922-7 21557001

[33] Williams J, Wake M, Hesketh K, Maher E, Waters E. Health-related quality of life of overweight and obese children. JAMA. 2005;293:70–6. 15632338 10.1001/jama.293.1.70

[34] Bisegger C, Cloetta B, von Rueden U, Abel T, Ravens- Sieberer U. Health-related quality of life: Gender differences in childhood and adolescence. Soz Praventivmed. 2005;50:281–91. 16300172 10.1007/s00038-005-4094-2

[35] Björkqvist K, Lagerspetz KMJ, Kaukiainen A. Do girls manipulate and boys fight? Developmental trends in regard to direct and indirect aggression. Aggress Behav. 1992;18:117–27.

[36] Waters E, Doyle J, Wolfe R, Wright M, Wake M, Salmon L. Influence of parental gender and self-reported health and illness on parent-reported child health. Pediatrics. 2000;106:1422–8. 11099598 10.1542/peds.106.6.1422

[37] Landgraf JM, Abetz L. Influences of sociodemographic characteristics on parental reports of children’s physical and psychosocial well-being: Early experiences with the Child Health Questionnaire In: Drotar D, editor. Measuring Health-Related Quality of Life in Children and Adolescents: Implications for Research and Practice. Mahwah: Lawrence Erlbaum Associates; 1998.

[38] Wilkins AJ, O’Callaghan MJ, Najman JM, Bor W, Williams GM, Shuttlewood G. Early childhood factors influencing health-related quality of life in adolescents at 13 years. J. Paediatr Child Health. 2004;40:102–9. 15009573 10.1111/j.1440-1754.2004.00309.x

[39] Botterweck A, Frenken F, Janssen S, Rozendaal L, de Vree M, Otten Fl. Feasibility, reliability and validity of the new measurements in the Dutch Health Interview Survey 2001 [Plausibiliteit nieuwe metingen algemene gezondheid en leefstijlen 2001] Voorburg/Heerlen: Statistics Netherlands; 2003.

[40] Norman GR, Sloan JA, Wyrwich KW. Interpretation of changes in health-related quality of life: The remarkable universality of half a standard deviation. Med Care. 2003;41(5):582–92. 12719681 10.1097/01.MLR.0000062554.74615.4C

[41] Hafkamp-de Groen E, Mohangoo AD, Landgraf JM, de Jongste JC, Duijts L, Moll HA, et al The impact of preschool wheezing patterns on health-related quality of life at age 4 years. Eur Respir J. 2013;41(4):952–9. 10.1183/09031936.00015712 22790911

