# Correction: Determinants of Health-Related Quality of Life in School-Aged Children: A General Population Study in the Netherlands

**DOI:** 10.1371/journal.pone.0134650

**Published:** 2015-07-30

**Authors:** Marieke Houben-van Herten, Guannan Bai, Esther Hafkamp, Jeanne M. Landgraf, Hein Raat

In Table 3, all values for the determinant “Siblings in household” are incorrect. The authors have provided a correct Table 3 here.

**Table 3:** Bivariate associations with CHQ-score and effect sizes based on interview administration methods.

Variable	Levels	PhS		PsS	
		mean (F-value, p-value)	sd *effect size*	mean (F-value, p-value)	sd *effect size*
**Gender**	**Male**	57.04	6.22	52.68	6.66
	**Female**	56.95	6.04	53.36	6.29
		(<1, .51)	*0*.*01*	(22.93, < .001)*	*0*.*10*
**Age**	**4**	56.63	6.47	53.30	6.12
	**5**	56.86	6.33	53.75	6.17
	**6**	57.42	5.90	53.06	6.28
	**7**	57.03	6.09	53.21	6.54
	**8**	57.09	5.92	52.49	6.64
	**9**	57.20	5.96	52.65	6.73
	**10**	56.73	6.33	52.70	6.78
	**11**	57.02	6.01	52.88	6.58
		(<2, .09)	*0*.*12*	(4.26, < .001)*	*0*.*19*
**Ethnicity**	**Native Dutch people**	57.15	6.11	53.10	6.42
	**Immigrants, Western**	56.50	6.52	52.68	6.72
	**Immigrants, Non-western**	56.35	6.31	52.68	6.60
		(9.13, < .001)*	*0*.*12*	(<2.5, .08)	*0*.*06*
**Urbanisation rate**	**Very high**	56.58	6.17	52.89	6.45
	**High**	56.96	6.09	53.04	6.55
	**Moderately high**	56.98	6.29	52.89	6.46
	**Low**	57.18	6.09	52.99	6.48
	**Very low**	57.32	5.98	53.32	6.49
		(2.82, .02)*	*0*.*12*	(<1, .44)	*0*.*07*
**Single parent family**	**Two parent family**	57.05	6.09	53.24	6.38
	**Single parent family**	56.60	6.47	50.95	7.07
		(4.01, .04)*	*0*.*07*	(96.81, < .001)*	*0*.*32* ^*a*^
**Siblings in household**	**Only child**	56.65	6.34	52.53	6.80
	**Has 1 brother or sister**	56.93	6.18	53.00	6.42
	**Has more brothers/sisters**	57.19	6.02	53.15	6.49
		(3.06, .05)*	*0*.*04*	(3.11, .04)*	*0*.*09*
**Working situation Parents**	**Both parents work**	57.17	6.01	53.13	6.35
	**One parent works**	56.90	6.32	53.09	6.48
	**Parents do not work**	55.53	6.98	51.63	7.25
		(15.62, < .001)*	*0*.*24* ^*a*^	(11.78, < .001)*	*0*.*21* ^*a*^
**Highest parental educational level**	**Low**	56.63	6.20	52.20	6.87
	**Medium**	57.19	6.16	52.96	6.52
	**High**	57.02	5.95	53.52	6.22
		(<2, .17)	*0*.*09*	(10.30, < .001)*	*0*.*19*
**Low household income**	**No**	57.04	6.10	53.17	6.39
	**Yes**	56.80	6.20	52.37	6.82
		(<1.5, .27)	*0*.*04*	(12.71, < .001)*	*0*.*12*
**Parents' smoking behaviour**	**Both parents smoke**	57.05	6.16	52.82	6.49
	**One parent smokes**	56.75	6.36	52.71	6.59
	**Parents do not smoke**	57.12	6.08	53.22	6.38
		(<2.5, .08)	*0*.*06*	(4.84, .01)*	0.08
**BMI child**	**Normal weight**	57.06	6.08	53.12	6.46
	**Overweight**	56.68	6.33	52.90	6.57
	**Obese**	55.86	6.31	51.49	7.02
		(5.37, .005)*	*0*.*19*	(7.65, < .001)*	*0*.*23* ^*a*^
**Nr of chronic conditions**	**None**	57.77	5.47	53.27	6.25
	**1**	55.04	6.96	52.41	6.98
	**2 or more**	50.36	8.27	50.80	7.92
		(341.50, < .001)*	*0*.*90* ^c^	(33.19, < .001)*	*0*.*31* ^*a*^
**Nr of behavioural /learning disorders**	**None**	57.05	6.02	53.45	6.21
	**1**	56.68	6.93	48.58	7.41
	**2 or more**	54.33	8.94	44.42	7.17
		(8.63, < .001)*	*0*.*30* ^a^	(237.35, < .001)*	*1*.*26* ^c^
**Number of acute health complaints**	**None**	58.44	4.79	53.86	6.07
	**1**	55.93	6.45	52.48	6.63
	**2**	53.70	7.59	51.53	7.25
	**3 or more**	50.51	8.67	50.94	7.58
		(353.60, < .001)*	*0*.*92* ^c^	(52.07, < .001)*	*0*.*36* ^a^
**Visited a GP last 14 days**	**No**	57.35	5.77	53.02	6.43
	**Yes**	52.41	8.49	52.84	7.20
		(367.28, < .001)*	*0*.*58* ^b^	(<1, .49)	*0*.*03*
**Visited medical specialist last 14 days**	**No**	57.15	5.97	53.05	6.44
	**Yes**	52.65	8.57	52.06	7.69
		(146.70, < .001)*	*0*.*53* ^b^	(6.69, .01)*	*0*.*13*
**Used prescription medicines last 14 days**	**No**	57.60	5.60	53.16	6.31
	**Yes**	53.32	7.74	52.14	7.42
		(519.34, < .001)*	*0*.*55* ^b^	(26.03, < .001)*	*0*.*14*
**Used non pre-scription medicines last 14 days**	**No**	57.86	5.39	53.24	6.34
	**Yes**	54.77	7.27	52.41	6.83
		(441.49, < .001)*	*0*.*42* ^a^	(27.85, < .001)*	*0*.*12*
**Hospitalization last year**	**No**	57.07	6.07	53.02	6.47
	**Yes**	52.88	8.03	52.45	7.62
		(64.39, < .001)*	*0*.*52* ^b^	(<1.5, .28)	*0*.*07*

Effect sizes are highest vs. lowest mean CHQ-score.

a = small difference

b = moderate difference

c = large difference.

# Data were calculates using US based weights and are provided for illustrative purposes. Not for general use.

* Statistically significant.
