# Supplementary material for: Determinants of Health-Related Quality of Life in School-Aged Children: A General Population Study in the Netherlands
Source: PLoS One. 2015 May 1;10(5):e0125083. doi: 10.1371/journal.pone.0125083 (PMC4416795; doi:10.1371/journal.pone.0125083)
Supplement: S1 Table — (DOCX) [file pone.0125083.s001.docx]

| **S1 Table. Illustration of age-gender groupings using interview administration.^a^** | |
| --- | --- |
|  |  |

| Parameter |  | Age-gender group | | | | | | | | | | |
| --- | --- | --- | --- | --- | --- | --- | --- | --- | --- | --- | --- | --- |
|  |  | 4-5 years | | 6-7 years | | 8-9 years | | 10-11 years | | Total | | Total |
|  |  | Boys | Girls | Boys | Girls | Boys | Girls | Boys | Girls | Boys | Girls | Total |
| PF | Mean | 94.3 | 93.9 | 94.7 | 95.3 | 95.3 | 95.2 | 94.4 | 93.9 | 94.7 | 94.6 | 94.6 |
|  | 95% CI | 93.4 - 95.2 | 92.9 - 95.0 | 93.8 - 95.6 | 94.4 - 96.1 | 94.5 - 96.0 | 94.3 - 96.0 | 93.5 - 95.3 | 92.9 - 94.8 | 94.2 - 95.1 | 94.1 - 95.0 | 94.3 - 94.9 |
|  | Sample N | 1420 | 1300 | 1355 | 1328 | 1334 | 1291 | 1314 | 1303 | 5423 | 5222 | 10645 |
| REB | Mean | 97.0 | 97.1 | 95.4 | 96.5 | 94.2 | 96.8 | 94.2 | 96.7 | 95.3 | 96.7 | 96.0 |
|  | 95% CI | 96.4 - 97.7 | 96.3 - 97.9 | 94.5 - 96.4 | 95.7 - 97.3 | 93.2 - 95.2 | 95.9 - 97.6 | 93.2 - 95.3 | 95.9 - 97.4 | 94.8 - 95.7 | 96.4 - 97.1 | 95.7 - 96.3 |
|  | Sample N | 1418 | 1299 | 1352 | 1329 | 1335 | 1289 | 1313 | 1301 | 5418 | 5218 | 10636 |
| RF | Mean | 95.0 | 94.7 | 95.2 | 95.4 | 95.9 | 96.3 | 95.4 | 95.2 | 95.4 | 95.4 | 95.4 |
|  | 95% CI | 94.1 - 95.5 | 93.7 - 95.7 | 94.2 - 96.1 | 94.4 - 96.4 | 95.1 - 96.8 | 95.4 - 97.1 | 94.5 - 96.4 | 94.2 - 96.2 | 94.9 - 95.8 | 94.9 - 95.9 | 95.1 - 95.7 |
|  | Sample N | 1419 | 1299 | 1352 | 1330 | 1336 | 1290 | 1314 | 1301 | 5421 | 5220 | 10641 |
| BP | Mean | 85.7 | 85.5 | 87.0 | 84.7 | 85.7 | 85.7 | 84.7 | 83.7 | 85.8 | 84.9 | 85.3 |
|  | 95% CI | 84.6 - 86.7 | 84.4 - 86.5 | 86.0 - 88.1 | 83.6 - 58.9 | 84.6 - 86.8 | 84.7 - 86.7 | 83.5 - 85.8 | 82.6 - 84.8 | 85.2 - 86.3 | 84.4 - 85.4 | 85.0 - 85.7 |
|  | Sample N | 1420 | 1300 | 1354 | 1330 | 1336 | 1290 | 1312 | 1303 | 5422 | 5223 | 10645 |
| BE | Mean | 69.3 | 71.3 | 69.0 | 73.1 | 69.3 | 72.7 | 69.5 | 73.6 | 69.3 | 72.7 | 71.0 |
|  | 95% CI | 68.4 - 70.1 | 70.5 - 72.2 | 68.1 - 70.0 | 72.2 - 74.0 | 68.4 - 70.3 | 71.7 - 73.6 | 68.5 - 70.5 | 72.7 - 74.5 | 68.8 - 69.8 | 72.2 - 73.1 | 70.6 - 71.3 |
|  | Sample N | 1400 | 1287 | 1339 | 1319 | 1324 | 1279 | 1299 | 1292 | 5362 | 5177 | 10539 |
| MH | Mean | 83.9 | 83.0 | 82.0 | 82.4 | 80.3 | 80.5 | 81.3 | 80.9 | 81.9 | 81.7 | 81.8 |
|  | 95% CI | 83.1 - 84.7 | 82.2 - 83.8 | 81.1 - 82.9 | 81.5 - 83.3 | 79.4 - 81.2 | 79.6 - 81.5 | 80.3 - 82.2 | 80.0 - 81.8 | 81.4 - 82.3 | 81.3 - 82.1 | 81.5 - 82.1 |
|  | Sample N | 1401 | 1283 | 1345 | 1316 | 1323 | 1282 | 1300 | 1293 | 5369 | 5174 | 10543 |
| SE | Mean | 83.2 | 84.1 | 81.0 | 82.7 | 79.4 | 80.0 | 78.1 | 79.7 | 80.5 | 81.6 | 81.0 |
|  | 95% CI | 82.5 - 83.9 | 83.4 - 84.9 | 80.2 - 81.7 | 81.9 - 83.4 | 78.6 - 80.2 | 79.2 - 80.8 | 77.3 - 79.0 | 78.9 - 80.4 | 80.1 - 80.9 | 81.2 - 82.0 | 80.8 - 81.3 |
|  | Sample N | 1385 | 1273 | 1338 | 1321 | 1324 | 1287 | 1306 | 1299 | 5353 | 5180 | 10533 |
| GH | Mean | 84.9 | 86.5 | 85.6 | 87.5 | 84.9 | 86.1 | 84.5 | 86.5 | 85.0 | 86.6 | 85.8 |
|  | 95% CI | 83.8 - 85.9 | 85.6 - 87.5 | 84.6 - 86.7 | 86.5 - 88.4 | 83.8 - 85.9 | 85.0 - 87.2 | 83.4 - 85.6 | 85.5 - 87.5 | 84.4 - 85.5 | 86.1 - 87.1 | 85.4 - 86.1 |
|  | Sample N | 1420 | 1299 | 1355 | 1329 | 1334 | 1291 | 1314 | 1301 | 5423 | 5220 | 10643 |
| PE | Mean | 89.4 | 90.5 | 88.4 | 89.6 | 87.6 | 89.2 | 88.2 | 89.5 | 88.4 | 89.7 | 89.0 |
|  | 95% CI | 88.5 - 90.3 | 89.6 - 91.4 | 87.5 - 89.3 | 88.7 - 90.5 | 86.7 - 88.5 | 88.3 - 90.2 | 87.3 - 89.2 | 88.3 - 90.5 | 88.0 - 88.9 | 89.3 - 90.2 | 88.7 - 89.4 |
|  | Sample N | 1417 | 1299 | 1352 | 1330 | 1335 | 1290 | 1313 | 1301 | 5417 | 5220 | 10637 |
| PT | Mean | 92.0 | 91.7 | 91.9 | 92.6 | 92.5 | 92.7 | 91.7 | 93.5 | 92.1 | 92.6 | 92.3 |
|  | 95% CI | 91.0 - 93.1 | 90.5 - 92.8 | 90.7 - 93.1 | 91.4 - 93.8 | 91.5 - 93.6 | 91.5 - 93.9 | 90.6 - 92.8 | 92.4 - 94.5 | 91.5 - 92.6 | 92.0 - 93.2 | 91.9 - 92.7 |
|  | Sample N | 1416 | 1300 | 1351 | 1328 | 1335 | 1289 | 1314 | 1302 | 5416 | 5219 | 10635 |
| FA | Mean | 88.2 | 88.8 | 88.8 | 91.0 | 89.9 | 91.6 | 90.6 | 92.6 | 89.4 | 91.0 | 90.2 |
|  | 95% CI | 87.3 - 89.2 | 87.8 - 89.9 | 87.7 - 89.8 | 90.0 - 92.0 | 88.9 - 90.9 | 90.6 - 92.6 | 89.6 - 91.5 | 91.8 - 93.4 | 88.9 - 89.9 | 90.5 - 91.5 | 89.8 - 90.5 |
|  | Sample N | 1417 | 1297 | 1352 | 1328 | 1333 | 1289 | 1314 | 1301 | 5416 | 5215 | 10631 |
| FC | Mean | 77.8 | 77.5 | 75.6 | 77.5 | 74.3 | 74.8 | 73.1 | 75.1 | 75.2 | 76.2 | 75.7 |
|  | 95% CI | 76.8 - 78.7 | 76.5 - 78.6 | 74.6 - 76.6 | 76.5 - 78.5 | 73.3 - 75.4 | 73.8 - 75.8 | 72.1 - 74.2 | 74.1 - 76.1 | 74.7 - 75.8 | 75.7 - 76.7 | 75.4 - 76.1 |
|  | Sample N | 1419 | 1300 | 1355 | 1329 | 1335 | 1291 | 1313 | 1302 | 5422 | 5222 | 10644 |
| CH | Mean | 59.0 | 57.6 | 58.4 | 56.8 | 57.0 | 56.4 | 56.8 | 54.7 | 57.8 | 56.4 | 57.1 |
|  | 95% CI | 58.0 - 60.0 | 56.6 - 58.6 | 57.4 - 59.5 | 55.9 - 57.7 | 56.0 - 58.1 | 55.5 - 57.3 | 55.7 - 57.8 | 53.8 - 55.5 | 57.3 - 58.4 | 55.9 - 56.8 | 56.8 - 57.5 |
|  | Sample N | 1421 | 1299 | 1355 | 1330 | 1335 | 1290 | 1313 | 1303 | 5424 | 5222 | 10646 |
| PhS | Mean | 55.5 | 55.4 | 56.2 | 56.1 | 56.4 | 56.3 | 55.9 | 55.6 | 56.0 | 55.9 | 55.9 |
|  | 95% CI | 55.0 - 56.1 | 54.9 - 56.0 | 55.7 - 56.7 | 55.6 - 56.6 | 55.9 - 56.8 | 55.8 - 56.7 | 55.4 - 56.4 | 55.1 - 56.1 | 55.8 - 56.3 | 55.6 - 56.1 | 55.8 - 56.1 |
|  | Sample N | 1355 | 1249 | 1313 | 1295 | 1300 | 1261 | 1276 | 1275 | 5244 | 5080 | 10324 |
| PsS | Mean | 53.2 | 53.5 | 52.1 | 53.3 | 51.5 | 52.5 | 51.6 | 52.9 | 52.1 | 53.1 | 52.6 |
|  | 95% CI | 52.8 - 53.6 | 53.1 - 53.9 | 51.7 - 52.5 | 52.9 - 53.7 | 51.0 - 52.0 | 52.1 - 52.9 | 51.1 - 52.1 | 52.5 - 53.3 | 51.9 - 52.3 | 52.9 - 53.3 | 52.4 - 52.7 |
|  | Sample N | 1355 | 1249 | 1313 | 1295 | 1300 | 1261 | 1276 | 1275 | 5244 | 5080 | 10324 |

a. For illustration purposes only. Not intended for data analysis or interpretation. Contact licensing@healthactchq.com for further details.
